# Evaluation of Non-dental open source software in comparison to dental software in construction of digitally designed partial dentures frameworks

**DOI:** 10.1038/s41598-025-12403-x

**Published:** 2025-07-28

**Authors:** Lina Samih Eldahmy, Marwa Ezzat Sabet, Fardos Nabil Rizk, Hebatallah Tarek Abdallah

**Affiliations:** 1 Oral & Maxillofacial Prosthodontics Department, British University in Egypt, El Sherouk City, 11837 Egypt; 2 Oral & Maxillofacial Prosthodontics Department, Ain Shams University and British University, Cairo, Egypt; 3 Department Head, Oral & Maxillofacial Prosthodontics Department, British University in Egypt, El Sherouk City, Egypt; 4 Oral & Maxillofacial Prosthodontics Department, Ain Shams University, Cairo, Egypt

**Keywords:** Digital dentistry, Digital dental software, Digital RPD design, CAD-CAM, Conventional RPD, Non-dental software, Dentistry, Biomedical materials

## Abstract

This study aimed to compare removable partial denture frameworks digitally designed using a non-dental software and two dental software programs. Frameworks were designed using Blender (Group A), Exocad (Group B), 3Shape (Group C), and a conventional fabrication method (Group D) on maxillary casts (*n* = 12 each ). Adaptation, retention, and surface roughness of manufactured frameworks were evaluated using Geomagic Control X software, a universal testing machine, and optical profilometry, respectively. Regarding adaptation results, 3Shape group had the lowest RMS value (1.79 mm **±** 0.13), whereas Blender group showed the highest RMS value (1.95 mm **±** 0.17). For retention, 3Shape group had the highest values (11.21 N **±** 0.07) while, Blender had the lowest retention (6.61 N **±** 1.13). Retention and adaptation tests showed statistically significant difference using ANOVA test (*P* ≤ 0.05). Surface roughness evaluation revealed no statistically significant differences among the four groups (*P* > 0.05). In conclusion, RPD frameworks designed using dental software demonstrated superior adaptation and retention compared to those designed with non-dental software. Dental software also outperformed the conventional technique in retention. Conventional frameworks showed better retention than those produced using non-dental software. No differences in surface roughness were found among the four groups.

## Introduction

Removable partial dentures (RPDs) have long been a reliable solution for managing partial edentulism. However, the conventional fabrication of RPDs remains complex, time-consuming, and prone to errors^1,2^. Key limitations of the traditional technique include physical impression steps and the potential for casting inaccuracies. Such inaccuracies often result from material distortion, human error, or patient-related factor as patient discomort^3^.

Since the emergence of digital dentistry in the 1970 s, CAD-CAM technology has been increasingly employed to overcome the limitations of traditional metal RPD frameworks^4^. This technology has evolved from simple milling processes to fully digital workflows for RPD design and production^5^. Intraoral scanners now enable highly accurate digital impressions, reducing patient discomfort and streamlining clinical steps. These scans are processed by specialized software to design precise frameworks. To further address the casting inaccuracies inherent in conventional methods, digital manufacturing techniques —such as subtractive milling and additive 3D printing—enable the accurate fabrication of metal frameworks^6^.

A complete digital workflow allows clinicians and dental technicians to perform digital surveying, framework design, and production entirely within a virtual environment. This approach simplifies communication between the clinic and the laboratory, reduces manual procedures, and supports rapid prototyping and adjustments^7,8^.

The concept of computer-assisted design for RPDs first emerged in 1985. At that time, the absence of reliable oral and laboratory scanning technologies required manual entry of information about undercuts on abutment teeth. Notably, expert systems developed in 1987 integrated artificial intelligence to automate the RPD design process, selecting appropriate abutment teeth and guiding the design based on clinical examination data^9^.

Today, RPD frameworks can be designed using either specialized dental software or non-dental software platforms. Open-source software such as Blender has gained popularity among dentists, designers, and technicians due to its cost-effectiveness. Dental add-ons and modules have been developed for Blender, enabling it to produce various dental prostheses and integrating it with implant and tooth libraries, making it comparable in functionality to dedicated dental software.

In recent years, Blender has been successfully applied to design diverse dental prostheses and surgical guides with promising results, including accurate stackable surgical guides, frameworks for full-arch implant prostheses, crowns, and bridges. However, studies specifically evaluating its performance for RPD framework design remain limited^10^.

The objective of this study was to design RPD frameworks using non-dental open-source software (Blender), two dental software programs (Exocad and 3Shape), and a conventional fabrication method, and to compare the resulting frameworks in terms of adaptation, retention, and surface roughness. The null hypothesis stated that no significant differences would be found among the three digital design software programs with respect to these parameters.

## Materials and methods

All information regarding the manufacturing process and equipment is provided in Table 1. Based on data from similar studies evaluating RPD framework retention, the response difference of the matched pairs was assumed to follow a normal distribution with a standard deviation of 0.17. Assuming a true mean difference of 0.2, a sample size of nine frameworks per group was required to achieve 85% power for rejecting the null hypothesis at a significance level of 0.05 (Type I error). To account for potential dropouts and ensure adequate statistical power, the sample size was increased to 12 frameworks per group, resulting in a total of 48 RPD frameworks^11^.

**Table 1:** Equipment and materials used.

Name	Machinery	Material/software
Selective laser melting (SLM)	**SLM 3D printing** **machine (VM 120;** **VULCANTECH GmbH)**	**Co-Cr powder (Starbond** **Easy Powder 30; Schefner** **dental alloys**,** GmbH)**
Universal testing machine	**Bluehill Lite; Instron Instruments** **(Model 3345; Instron Instruments Ltd.**,** USA)**	**(Bluehill Lite; Instron Instruments) software**
Digital microscope	**(U500x Digital Microscope**,** Guangdong**,** China)**	**WSxM software (Ver 5 develop 4.1**,** Nanotec**,** Electronica**,** SL)**

An educational maxillary cast with a bilateral bounded edentulous area (Kennedy Class III modification 1) was used in this study. Mouth preparation was performed on the distal third of the first right and left premolars and the mesial third of the right and left second molars to create occlusal rests. Beading was performed by scraping the cast to a depth and width of 0.5 mm along the borders of the maxillary major connector.

**Fig. 1:**
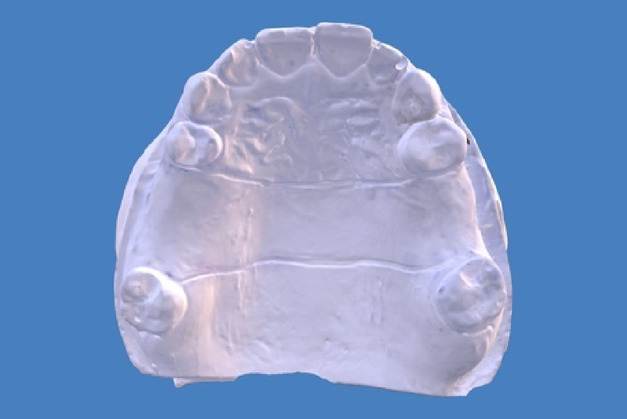
Virtual model of the stone cast.

The cast was scanned using a desktop scanner (E2; 3Shape A/S) to generate a virtual model (Fig. 1). An STL file of the prepared cast was produced and used to fabricate 3D-printed replica models for further testing across all four study groups.

All frameworks were designed by the same operator using the STL file of the virtual cast. The frameworks were divided into four groups: Blender, Exocad, 3Shape, and a conventional fabrication group (*n* = 12). The operator had the same level experience with all software programs to ensure reproducibility and reduce bias.

The design process was similar across all software programs. First, the cast was prepared by trimming the base and aligning it to the correct occlusal plane. In Exocad and 3Shape, the master cast was then surveyed, and blockout wax was adjusted to eliminate undesired undercuts, creating a virtual modified master cast. In Blender, the articulator and virtual facebow modules were used to align the model on the mounting table. An offset model was then created to compensate for potential printing errors, using an offset of 0.2 mm and a voxel size of 0.2 mm as recommended for optimal surface quality. The offset model was generated 200 microns larger than the original.

**Fig. 2:**
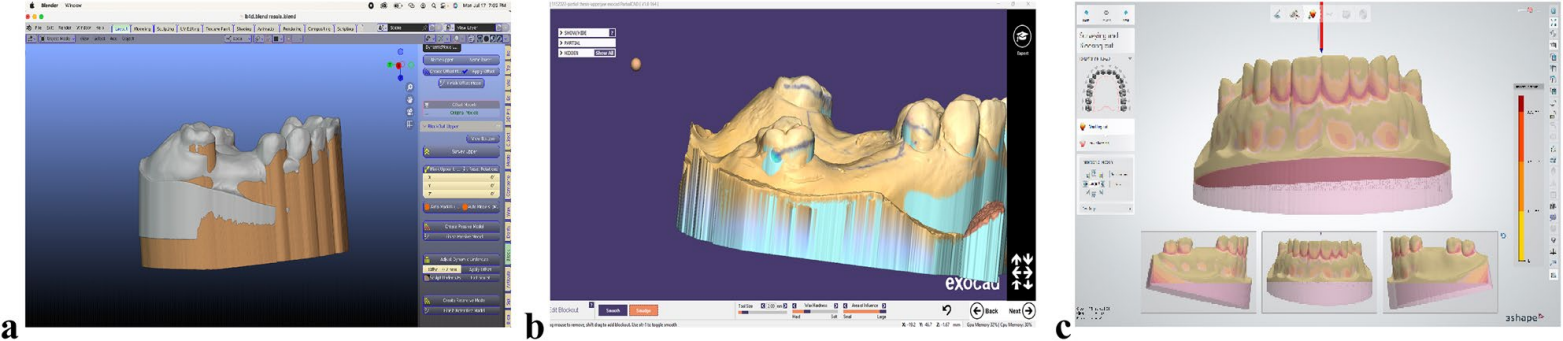
Block out step. **A**, Blender software. **B**, Exocad software. **C**, 3Shape software.

During the surveying step, all software programs automatically provided a zero-tilt orientation of the cast as the default reference for the path of insertion. Although each software allows the cast to be tilted in any direction to adjust undercuts and guiding planes, this step was standardized in the present study by retaining the default zero-tilt position. This ensured consistent undercuts, proper guiding planes, and a uniform path of insertion across all groups. After blockout verification, a passive model was created in Blender, and the sculpt tool was used with the dynamic undercut offset set to − 0.3 mm (Fig. 2a–c).

The framework design in all software programs followed the standard steps provided by each platform. A middle palatal strap major connector was selected to connect the denture bases bilaterally. Retentive and reciprocal clasp arms were designed on three abutments, excluding the right first premolar. The 1.5 mm retentive arm was positioned buccally above the survey line, with a tapered 1 mm tip extending below the survey line to engage a 0.3 mm undercut. The clasp arm was joined to the occlusal rest and minor connector to form a unified framework. Likewise, a 1.5 mm reciprocal arm was designed on the palatal surface of each abutment and connected to the minor connector and occlusal rest.

In Blender, these design steps were completed in Edit mode. Sculpt mode was then used to smooth and adjust the framework as needed. The Re-mesh and cutting the fitting surface tools were applied to ensure uniform voxel distribution and a smooth surface finish. In Exocad and 3Shape, the framework design was similarly refined using sculpting tools to smooth rough areas and remove sharp edges. After finalizing the design in each software program, an STL file was generated for fabrication.

**Fig. 3:**
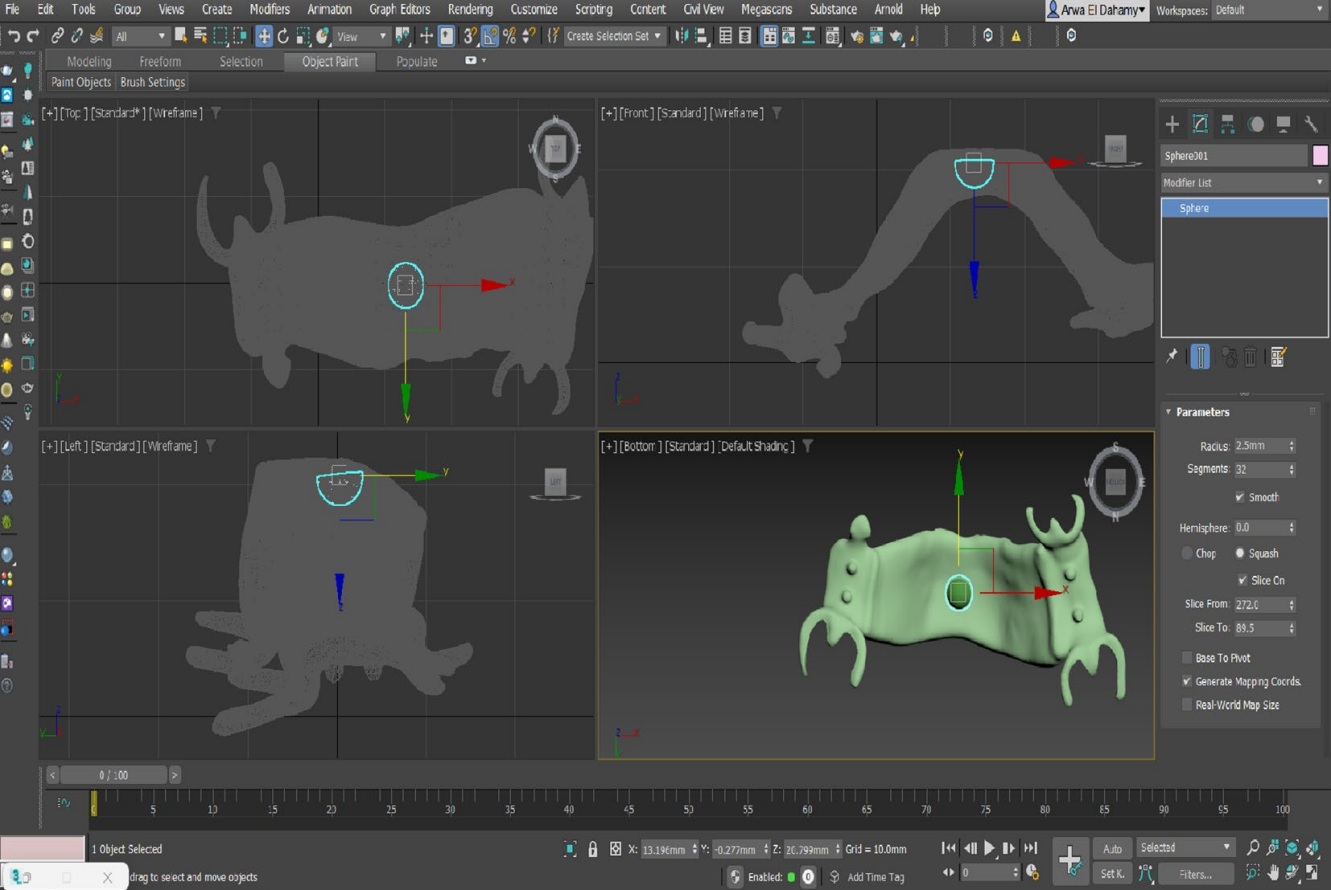
Creating the geometric center.

**Fig. 4:**
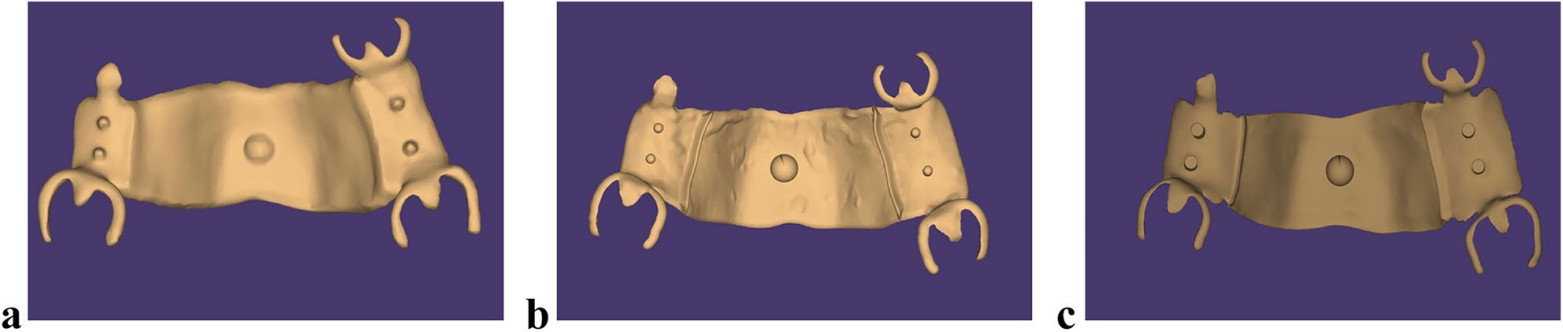
Final design of RPD framework. **a**, Blender software. **a**, Exocad software. **c**, 3Shape software.

The designed RPD STL files from all software programs were imported into AUTODESK 3DS MAX 2022 to determine the center of each framework and generate a central sphere (Fig. 3). This sphere was used to fix a metal ring centrally positioned between 5 and 6 bilaterally for the retention test conducted later^12,13^. The final STL files (Fig. 4a–c) were then imported into Materialise Magics 22.03 software to add supports for the printing process. The frameworks were fabricated using selective laser melting, after which the supports were carefully removed. Each framework was then scanned using the same 3Shape desktop scanner, with scanning spray applied as a matting agent to reduce light reflection caused by the alloy’s polished surface. The spray was applied at a standardized distance and angle to ensure a thin, uniform, and homogeneous coating on all surfaces. Each framework was visually inspected to confirm an even, opaque, matte layer, minimizing the risk of artifacts that could affect adaptation measurements.

**Fig. 5:**
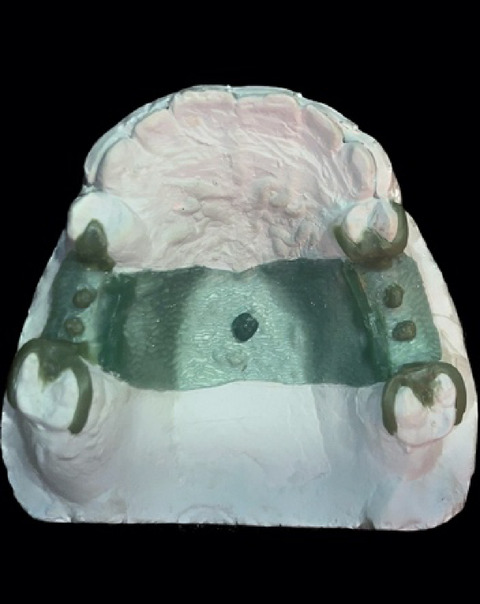
Conventional Wax pattern.

In the conventional group, the cast was duplicated using a silicone-based duplication material (REPLISIL 22 N, dent-e-con, Germany), and twelve casts were produced using type IV dental stone (GC Fujirock^®^ EP OptiXscan, GC EUROPE, Belgium). All casts were poured on the same day and stored in a dry environment for 24 h. Each cast was first surveyed, and the path of insertion, desirable undercuts (matching the depth used in the digital groups), and undesirable undercuts were determined. The casts were then modified by blocking out the unwanted undercuts to produce a modified master cast. A refractory cast was made to begin the wax pattern and framework design. All design elements were performed in the same manner as the digital groups to maintain standardization, using the same operator. The geometric center of the framework was determined manually, and a wax sphere was created at the center, as was done with the digital designs (Fig. 5). The frameworks were produced using the lost-wax technique and metal casting. The finished frameworks were polished, leaving the fitting surface unpolished, and then scanned with the same scanner for adaptation evaluation.

Samples were coded by a second operator, and a third operator conducted the evaluation process to ensure blindness^14–16^.

The adaptation of each framework’s fitting surface was assessed using Geomagic Control X software for all study groups. The STL file of the model cast was designated as the “reference data,” while the framework’s fitting surface was designated as the “measured data.” The two files were aligned using the software’s best-fit alignment feature to ensure optimal superimposition of the cast and framework. A color map was then generated to visualize the adaptation by illustrating the distance between the framework’s fitting surface and the model cast. Additionally, numerical data were generated to quantify the adaptation as root mean square (RMS) error.

**Fig. 6:**
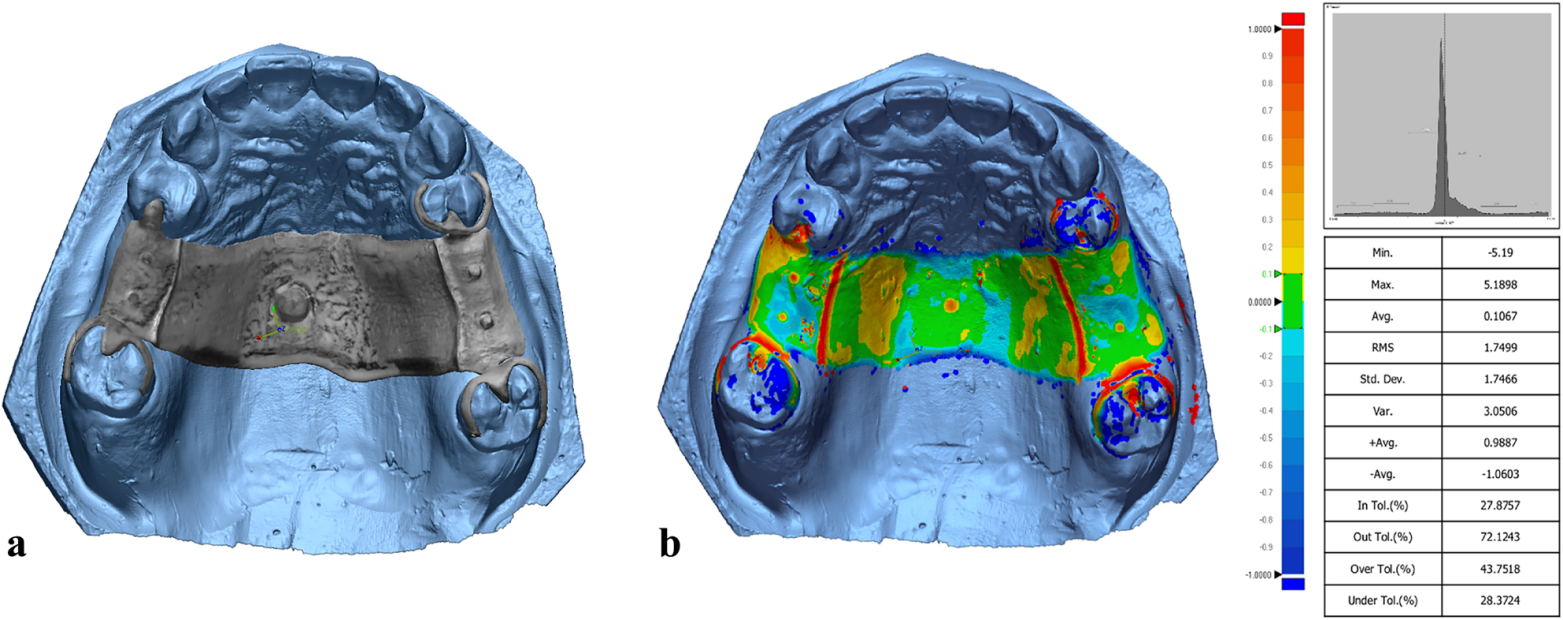
Adaptation evaluation using Geomagic software. a, superimposition of framework on the cast for adaptation evaluation. b, Color map showing adaptation results.

In digital dentistry, RMS error is a statistical measure that indicates the average difference between two 3D surfaces (STL files). A lower RMS error means the manufactured framework closely matches the digital design, indicating a precise fit, while a higher RMS error suggests greater deviation (Fig. 6a, b)^5,17^.

Retention tests were performed using a universal testing machine (Bluehill Lite; Instron Instruments). Each partial denture was secured to the cast and mounted to the lower fixture of the testing machine using a 5 kN load cell. A metal ring fixed to the framework was attached to a metal hook suspended from the upper mobile part of the machine to maintain alignment with the loading axis and ensure even load distribution. Five vertical pull tests were performed for each framework, with force measured in newtons. The mean of the five tests was then calculated as the retention value^18,19^.

**Fig. 7:**
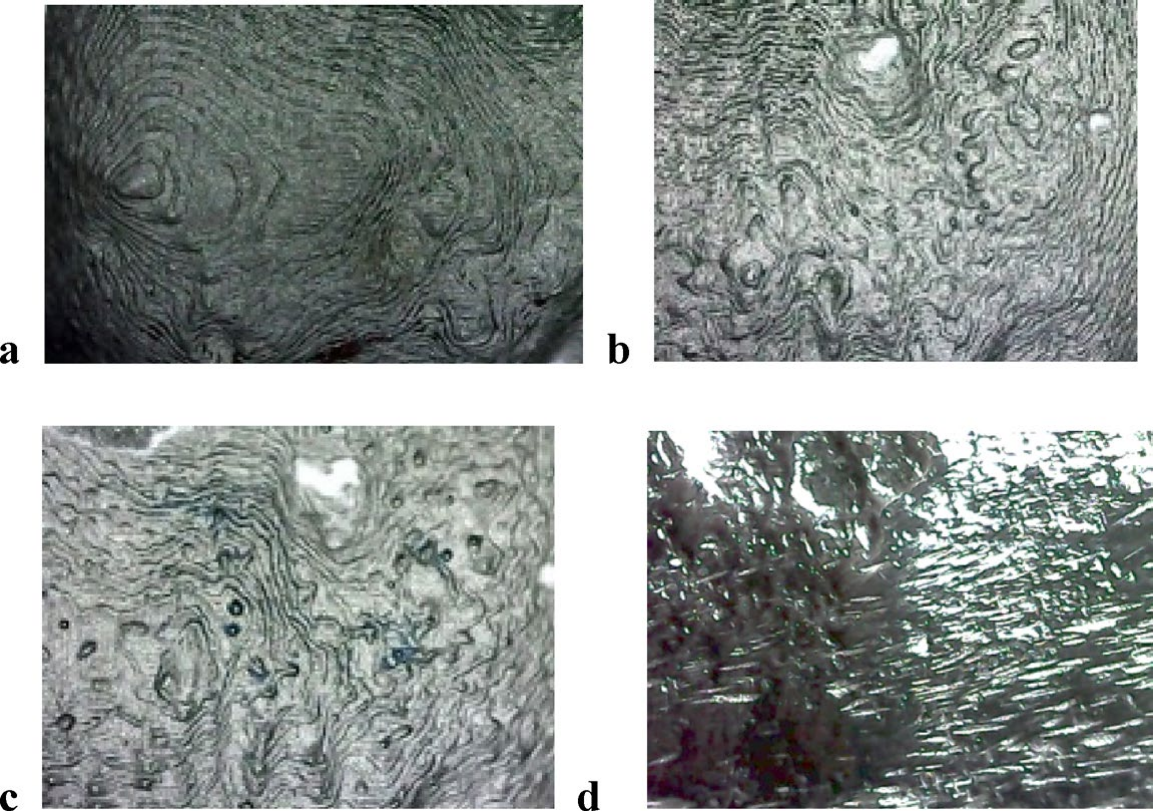
**a**, Cropped digital microscope image of RPD framework (Blender). **b**, Exocad framework. **c**, 3Shape framework. **d**, Conventional group framework.

**Fig. 8:**
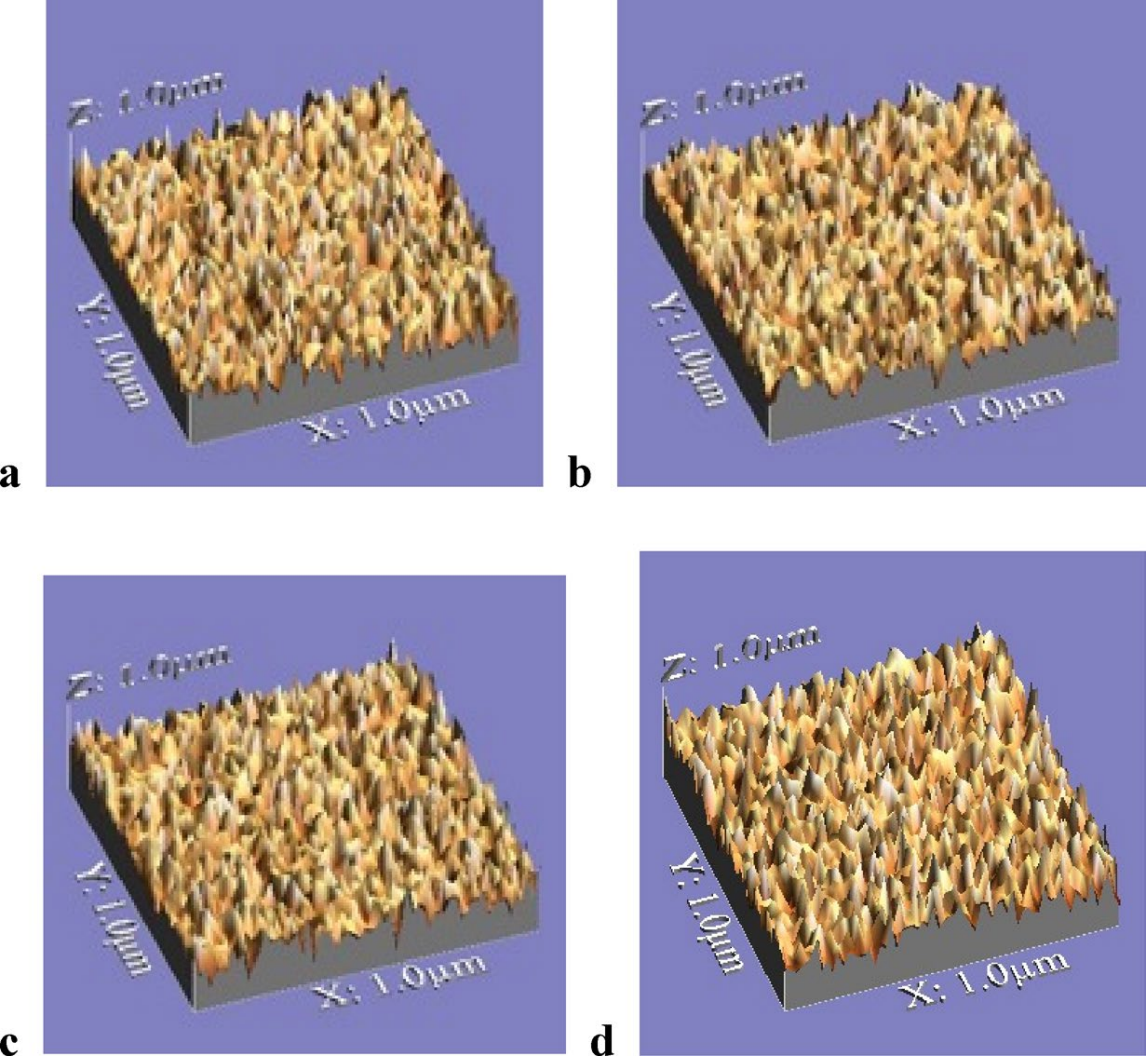
3D Geometry of fitting surface. **a**, Blender framework. **b**, Exocad framework. **c**, 3Shape framework. **d**, Conventional group framework.

Surface roughness was assessed using an optical method. Images were captured with a digital camera equipped with eight LED lamps for illumination, at maximum resolution and a fixed magnification of 90×. The WSxM software was used to analyze the cropped images (Fig. 7a–d). For each framework, three 3D images were taken: one from the middle region and two from the periphery, each covering an area of 10 μm × 10 μm (Fig. 8a–d). The WSxM software determined the average height (Ra) and maximum height, both reported in micrometers (µm)^20–23^.

Numerical data were reported as mean and standard deviation (SD) values. Data normality was verified using Shapiro–Wilk’s test, and variance homogeneity was assessed using Levene’s test. Statistical analyses were performed using IBM SPSS Statistics version 20 for Windows. ANOVA was used to compare the four groups, and pairwise comparisons were conducted with Tukey’s HSD post-hoc test when ANOVA results were significant. The level of significance was set at *P* ≤ 0.05.

## Results

Data are presented as mean and standard deviation values for all groups. This study’s results are shown in Tables (2–4).

### Framework adaptation (RMSD)

The results for framework adaptation, expressed as root mean square deviation (RMSD) values, showed that the 3Shape group had the lowest RMS error, indicating the best adaptation. The Exocad group exhibited higher deviation, followed by the conventional group, while the Blender group had the highest deviation and therefore the least adaptation. ANOVA revealed a statistically significant difference (*P* = 0.02). Tukey’s post-hoc test indicated a significant difference only between the Blender and 3Shape groups. No significant differences were found between Blender and Exocad, Blender and conventional, Exocad and 3Shape, Exocad and conventional, or 3Shape and conventional groups (Table 2).

**Table 2:** Mean, standard deviation, and ANOVA P-value of deviation values for framework adaptation in (mm**)** across four study groups.

	Mean	± SD	*P*-Value	F-Value
**Blender group**	**1.95** mm **(a)**	**0.17**	**0.02**	**3.8**
**Exocad group**	**1.85** mm **(a-b-c)**	**0.13**		
**3Shape group**	**1.79** mm **(b-c)**	**0.13**		
**Conventional group**	**1.89** mm **(a-b-c)**	**0.017**		

* SD: Standard deviation, P; Probability level, Significant difference (*P* ≤ 0.05). Different letters reflect statistically significant differences in framework adaptation between groups.

### Retention test

For retention, ANOVA results (*P* = 0.00001) showed that the 3Shape group frameworks achieved the highest retention values, followed by the Exocad group and the conventional group, while the Blender group showed the lowest retention. Tukey’s HSD post-hoc test revealed statistically significant differences between Blender and Exocad, Blender and 3Shape, and Blender and conventional groups. Significant differences were also found between the conventional group and Exocad, and between the conventional group and 3Shape. There was no significant difference between the Exocad and 3Shape groups. (Table 3).

**Table 3:** Mean, standard deviation, and ANOVA P-value of retention values in newtons for frameworks across four study groups.

Software Type	Mean	± SD	*P*-Value	F-Value
**Blender group**	**6.61** N **(a)**	**1.13**	**0.00001**	**49.37**
**Exocad group**	**10.77** N **(b)**	**1.73**		
**3Shape group**	**11.21** N **(b)**	**0.07**		
**Conventional group**	**8.94** N **(c)**	**0.05**		

* SD: Standard deviation, P; Probability level, Significant difference (*P* ≤ 0.05). Different letters reflect statistically significant differences in framework adaptation between groups.

### Surface roughness

For surface roughness, the Exocad and conventional group frameworks had the highest roughness values, while the 3Shape group showed lower values, and the Blender group showed the lowest. ANOVA indicated no statistically significant differences among the four groups (*P* = 0.3). (Table 4).

**Table 4:** Mean, standard deviation, and ANOVA P-value of surface roughness values in (µm) for frameworks across four study groups.

Software Type	Mean	± SD	*P*-Value	F-Value
**Blender group**	**0.24** μm	**0.04**	**0.3**	**1.26**
**Exocad group**	**0.26** μm	**0.02**		
**3Shape group**	**0.25** μm	**0.03**		
**Conventional group**	**0.26** μm	**0.004**		

* SD: Standard deviation, P; Probability level, Significant difference (*P* > 0.05).

## Discussion

The null hypothesis that no significant differences would exist among the three software programs was rejected. In this in vitro study, three different software programs (two dental and one non-dental) and a conventional technique were compared for designing RPD frameworks in terms of adaptation, retention, and surface roughness. This study assessed each software’s capability to produce a retentive, well-adapted, and adequately smooth metal framework. The in vitro design allowed for precise control and standardization, which is ideal for producing reliable, repeatable results, especially in comparative studies. Moreover, in vitro tests are considered more accurate because they can be replicated under consistent conditions, with the variable of interest being isolated^24^.

Framework design in all groups began by applying the basic principles for tooth-supported partial dentures. An Aker clasp was selected because it provides both retention and stabilization. A tripodal retention design was implemented due to the relatively short span of the edentulous region. Because molars withstand greater occlusal forces than premolars, the upper right premolar was used only for a rest seat to provide support, improving both aesthetics and biomechanics. The other three abutment teeth received a retentive clasp with a terminal end positioned to engage an undercut of approximately 0.3 mm (0.01 inches)^25,26^. In each software program, the same basic steps were followed: surveying and blockout, component design, and finalizing the framework. A middle palatal strap was chosen to provide rigidity while avoiding the bulk of bar-type connectors that could interfere with tongue movement and speech — the same design was applied in the conventional group.

Beading was performed before scanning the master cast to standardize the procedure across all groups and eliminate variables that could affect the results^27^.

### Adaptation

Statistical analysis showed a significant difference in framework adaptation among the four groups (*P* = 0.02). However, Tukey’s test indicated no significant difference between Exocad and 3Shape, aligning with previous studies evaluating denture base adaptation using these programs^28^. This may be due to their standardized workflows and reliable algorithms. A significant difference was found between Blender and 3Shape, which contrasts with a previous study on implant positioning accuracy using Blender/3D Slicer compared to Blue Sky software, which reported no differences^10^. This inconsistency may be explained by the greater complexity of RPD design compared to surgical guide design—specifically the creation of offset models, retentive areas, blockouts, and undercuts, which are critical for RPD adaptation and retention.

No significant difference was found between the conventional group and the three digital groups, which is consistent with other studies comparing the fit of conventional and CAD-CAM RPD frameworks^8,9,29^. This may be attributed to the simplicity of the design and the use of pre-programmed parameters (e.g., surveying, clasp dimensions) in digital workflows, which closely mimic conventional design principles and reduce variability.

### Retention

Retention testing revealed statistically significant differences among the groups (*P* = 0.00001). The 3Shape group showed the highest retention, followed by Exocad, then the conventional group, with Blender showing the lowest retention. Dental software simplifies the design process into clear, step-by-step workflows, minimizing human error and ensuring consistent undercuts and clasp adaptation. Although Blender can be adapted for dental use with specific modules, it lacks some built-in automation found in dental software. Manual sculpting in Blender can result in an irregular mesh that affects the shape of the framework’s undercuts and retentive components, increasing the risk of variation. Frequent re-meshing and voxel size adjustments are required to maintain surface uniformity, which can affect final adaptation and retention. In contrast, Exocad and 3Shape automate many of these steps, providing more predictable and standardized results. The threshold for RPD retention depends on clasp design, abutment undercut depth, material properties, and framework fit^30^.

These findings align with prior research showing that digital RPD frameworks typically achieve greater retention than conventional frameworks, likely due to the elimination of errors common in conventional impression and casting techniques^31^. On the contrary, another study done to evaluate retention of SLM clasps with casted clasp assembly which was digitally designed, found no statistical difference. This is likely because the groups were fabricated with the same digital data such that all the clasps had the same arm length, thickness, and cross-sectional area with the difference only in final fabrication method unlike the present study. Additionally, that study focused on clasp assemblies only, while complete frameworks introduce more factors, including major connectors, rest seats, and overall fit^31^.

### Surface roughness

Surface roughness results showed no significant differences among the groups, and all values fell within clinically acceptable ranges^32^. This may be because the same material and selective laser melting technique were used for all digital groups. The re-mesh step in Blender may account for its slightly lower surface roughness, as voxel size and mesh uniformity directly affect surface topography^33,34^. The conventional group also showed similar roughness to digital groups, which is consistent with a study done by Takaichi et al.^12^ who reported no significant difference in the surface roughness of clasp arms produced by SLM and traditional casting. Modern SLM machines can achieve comparable smoothness due to calibrated laser power, layer thickness, and scan strategies that minimize surface irregularities. Clinically acceptable roughness is generally ≤ 0.25 μm; higher values (> 0.30 μm) may increase plaque accumulation and patient discomfort, but all groups in this study remained within acceptable limits^32^.

A primary limitation of this study is the exclusive use of a single RPD design representing a bounded partially edentulous maxilla. These findings may not fully apply to other designs, particularly free-end saddle cases that present different biomechanical and retention challenges. Future studies should include a wider range of partial denture designs, especially distal extension cases, to more comprehensively evaluate framework adaptation and retention across diverse clinical scenarios.

From the previous results the following was concluded:

1. RPD frameworks digitally designed using specialized dental software (Exocad and 3Shape) exhibited superior adaptation and retention compared to those designed with non-dental open-source software (Blender).
2. Dental software outperformed the conventional fabrication technique in terms of retention.
3. Frameworks produced by the conventional technique showed better retention than those designed using non-dental software.
4. No difference in surface roughness evaluation were observed among the four groups, indicating that the manufacturing method and material had a greater influence on this parameter than the design software.

## Data Availability

The data used to support the results of this study are available from the corresponding author.
